# Supplementary figures and images for: Ambient temperature and mental health hospitalizations in Bern, Switzerland: A 45-year time-series study
Source: PLoS One. 2021 Oct 12;16(10):e0258302. doi: 10.1371/journal.pone.0258302 (PMC8509878; doi:10.1371/journal.pone.0258302)

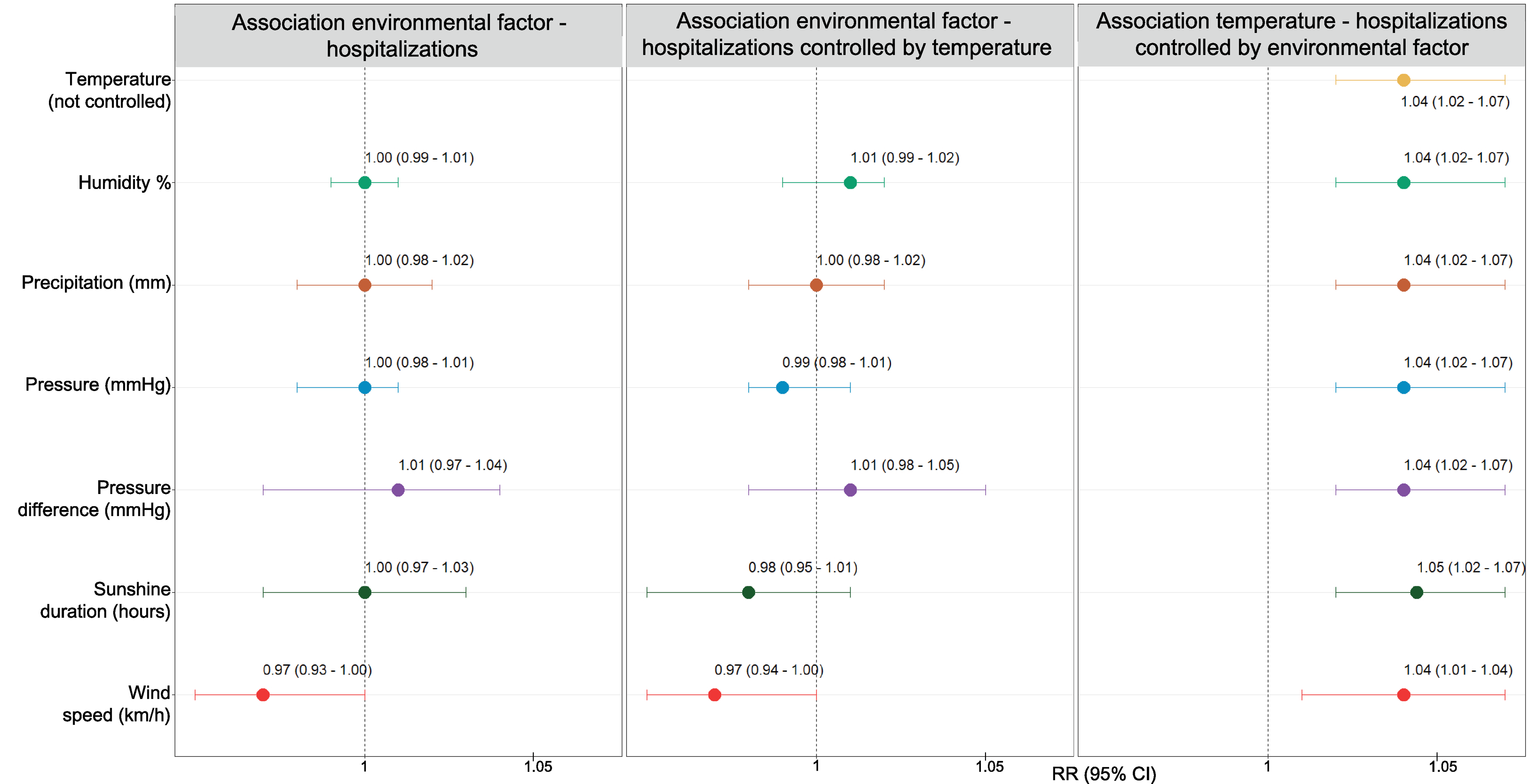

Supplement: S1 Fig — Null hypothesis is that there is no association (RR = 1), thus one can reject the null hypothesis when 95% confidence interval does not include 1. (TIF) [file pone.0258302.s001.tif]

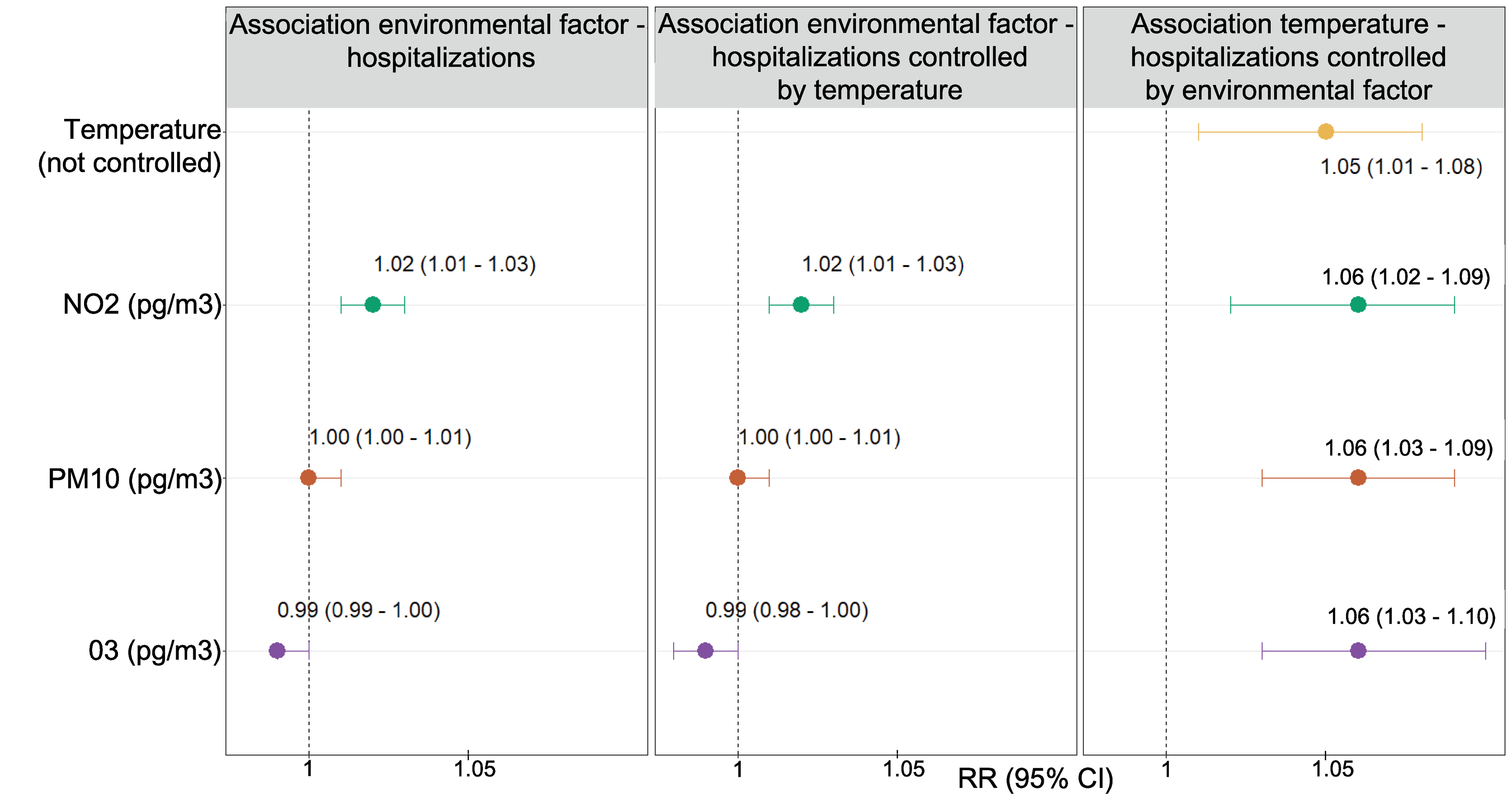

Supplement: S2 Fig — Null hypothesis is that there is no association (RR = 1), thus one can reject the null hypothesis when 95% confidence interval does not include 1. (TIF) [file pone.0258302.s002.tif]

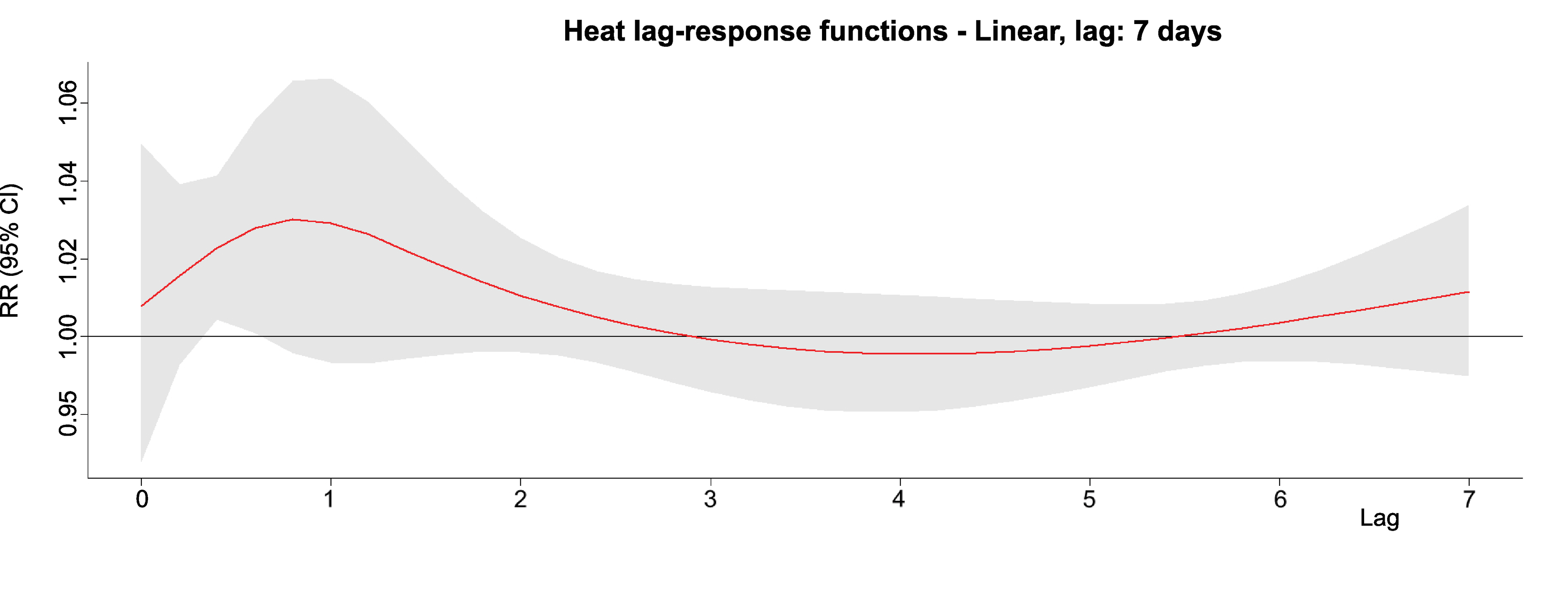

Supplement: S3 Fig — (TIF) [file pone.0258302.s003.tif]

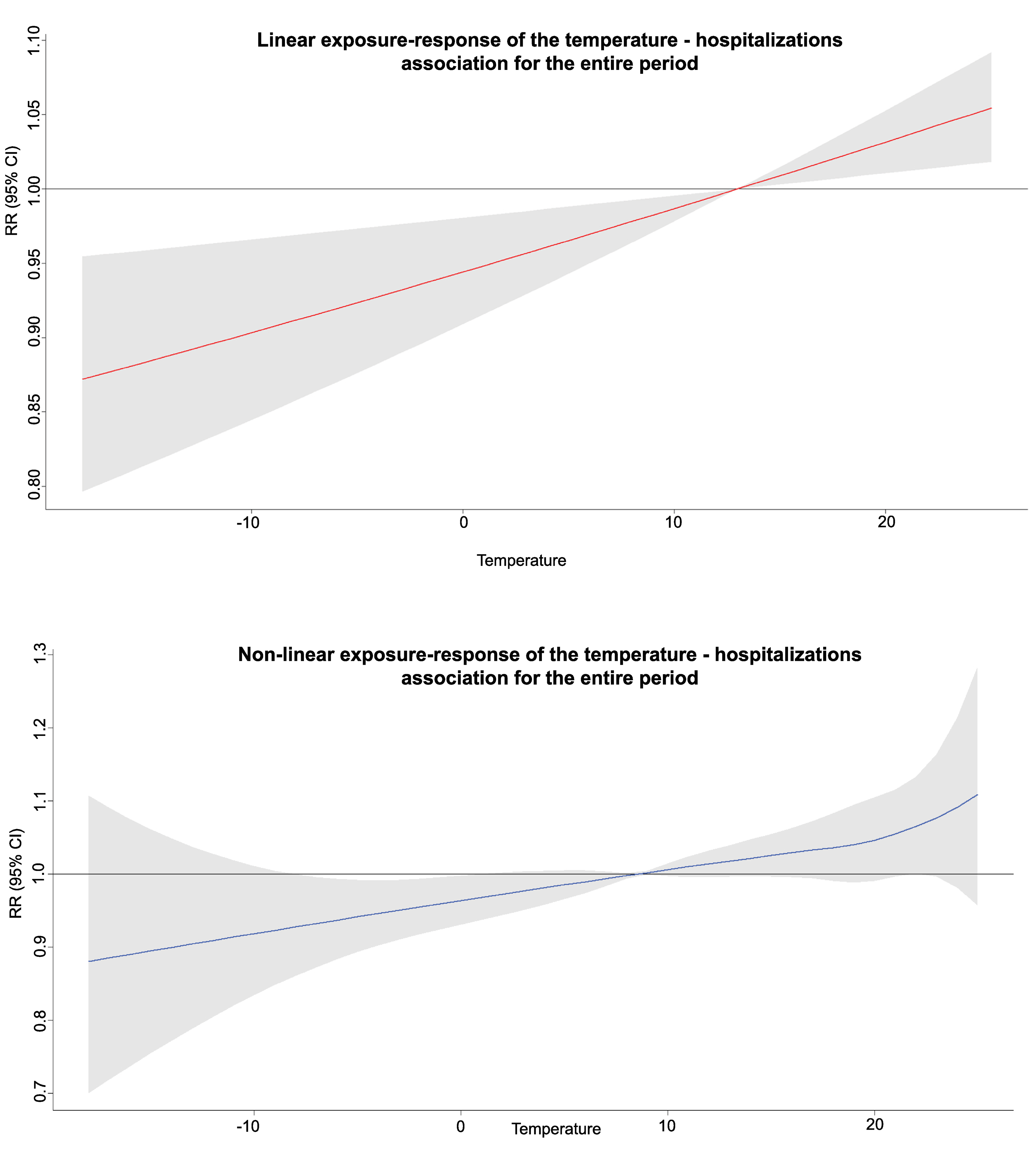

Supplement: S4 Fig — (TIF) [file pone.0258302.s004.tif]
