# Supplementary material for: Ambient temperature and mental health hospitalizations in Bern, Switzerland: A 45-year time-series study
Source: PLoS One. 2021 Oct 12;16(10):e0258302. doi: 10.1371/journal.pone.0258302 (PMC8509878; doi:10.1371/journal.pone.0258302)
Supplement: S1 Table — (DOCX) [file pone.0258302.s005.docx]

| **Environmental Variables (daily means)** | **Minimum** | **25th Percentile** | **Median** | **Mean** | **75th Percentile** | **Maximum** |
| --- | --- | --- | --- | --- | --- | --- |
| Humidity (%) | 35.0 | 70.6 | 78.8 | 77.9 | 85.8 | 100.00 |
| Precipitation (mm) | 0.0 | 0.0 | 0.0 | 2.8 | 2.7 | 90.3 |
| Pressure (mmHg) | 910.8 | 947.5 | 952.2 | 951.7 | 956.4 | 974.2 |
| Pressure difference | -26.6 | -2.5 | -0.1 | -0.0 | 2.4 | 22.6 |
| Sunshine duration (hours) | 0.0 | 0.4 | 3.6 | 4.7 | 8.1 | 14.8 |
| Wind speed (m/h) | 0.4 | 4.0 | 5.0 | 6.2 | 7.6 | 38.9 |
| NO2 (µg/m3)* | 10.5 | 38.6 | 48.0 | 49.1 | 58.1 | 120.0 |
| PM10 (µg/m3)* | 2.9 | 20.1 | 29.1 | 33.8 | 41.9 | 214.5 |
| O3 (µg/m3)* | 0.1 | 9.2 | 23.4 | 27.2 | 42.6 | 106.4 |
| **Extreme temperature events (May-September)** | **Number (%)** | | | | | |
| >= 2-days duration heatwave temperature ≥ 92.5^th^ percentile (19.1 °C) | 1148 (16.67) | | | | | |
| >= 2-days duration heatwave with temperature ≥ 97.5^th^ percentile (21.3 °C) | 354 (5.14) | | | | | |
| >= 3-days duration heatwave with temperature ≥ 92.5^th^ percentile (19.1 °C) | 1020 (14.81) | | | | | |
| >= 3-days duration heatwave with ≥ 97.5^th^ percentile (21.3 °C) | 274 (3.98) | | | | | |

*Data available only for the period 1991-2017.
