# Supplementary material for: Ambient temperature and mental health hospitalizations in Bern, Switzerland: A 45-year time-series study
Source: PLoS One. 2021 Oct 12;16(10):e0258302. doi: 10.1371/journal.pone.0258302 (PMC8509878; doi:10.1371/journal.pone.0258302)
Supplement: S2 Table — (DOCX) [file pone.0258302.s006.docx]

|  | **Study period 1973-1989** | **Study period 1990-2017** |
| --- | --- | --- |
| Total | 1.02 (0.97 - 1.08) | 1.05 (1.02 - 1.08) |
| Male | 1.00 (0.93 - 1.08) | 1.06 (1.02 -1.11) |
| Female | 1.04 (0.95 - 1.13) | 1.04 (1.00 - 1.08) |
| <65 years old | 1.00 (0.94 - 1.06) | 1.06 (1.02 - 1.09) |
| >=65 years old | 1.18 (1.02 - 1.38) | 1.0 (0.93 - 1.08) |
| Organic mental disorders (F00-F09) | 1.13 (0.92 - 1.38) | 0.94 (0.81 - 1.09) |
| Psychoactive substance use (F10-F19) | 0.95 (0.85 - 1.05) | 1.03 (0.97 - 1.10) |
| Schizophrenia (F20-29) | 1.06 (0.95 - 1.18) | 1.10 (1.04 - 1.17) |
| Mood disorders (F30-39) | 1.03 (0.88 - 1.21) | 1.05 (0.98 - 1.12) |
| Neurotic disorders (F40-59) | 1.09 (0.92 - 1.29) | 1.04 (0.96 - 1.12) |
| Adult personality disorders (F60-69) | 1.08 (0.86 - 1.34) | 0.99 (0.88 - 1.10) |
| Mental retardation (F70-79) | 0.88 (0.45 - 1.73) | 0.89 (0.65 - 1.20) |
| Developmental disorders (F80-98) | 1.15 (0.77 - 1.73) | 1.31 (1.08 - 1.60) |
