# Supplementary material for: Ambient temperature and mental health hospitalizations in Bern, Switzerland: A 45-year time-series study
Source: PLoS One. 2021 Oct 12;16(10):e0258302. doi: 10.1371/journal.pone.0258302 (PMC8509878; doi:10.1371/journal.pone.0258302)
Supplement: S3 Table — Null hypothesis is that there is no association (RR = 1), thus one can reject the null hypothesis when 95% confidence interval does not include 1. (DOCX) [file pone.0258302.s007.docx]

| **Duration (days)** | **Temperature (percentile)** | **RR** | **Lower CI** | **Upper CI** |
| --- | --- | --- | --- | --- |
| 2.00 | 92.50 | 1.04 | 0.93 | 1.15 |
| 2.00 | 97.50 | 1.04 | 0.83 | 1.29 |
| 3.00 | 92.50 | 1.05 | 0.95 | 1.17 |
| 3.00 | 97.50 | 1.06 | 0.82 | 1.36 |
