# Supplementary material for: Ambient temperature and mental health hospitalizations in Bern, Switzerland: A 45-year time-series study
Source: PLoS One. 2021 Oct 12;16(10):e0258302. doi: 10.1371/journal.pone.0258302 (PMC8509878; doi:10.1371/journal.pone.0258302)
Supplement: S4 Table — Null hypothesis is that there is no association (RR = 1), thus one can reject the null hypothesis when 95% confidence interval does not include 1. (DOCX) [file pone.0258302.s008.docx]

| Exposure-response dimension | Lag-response dimension | Lag | RR | CI Upper | CI Lower | QAIC |
| --- | --- | --- | --- | --- | --- | --- |
| Linear | Unconstraint | 3.00 | 1.04 | 1.01 | 1.07 | 56,004.17 |
| B-spline (50th, 90th percentile) | Unconstraint | 3.00 | 1.06 | 1.00 | 1.13 | 56,025.59 |
| B-spline (10th, 75th, 90th percentile) | Unconstraint | 3.00 | 1.05 | 0.99 | 1.10 | 56,028.76 |
| Linear | Strata lag0, lag1-3 | 3.00 | 1.05 | 1.02 | 1.08 | 56,002.42 |
| B-spline (50th, 90th percentile) | Strata lag0, lag1-3 | 3.00 | 1.06 | 1.00 | 1.13 | 56,018.49 |
| B-spline (10th, 75th, 90th percentile) | Strata lag0, lag1-3 | 3.00 | 1.07 | 1.00 | 1.13 | 56,021.48 |
| Linear | Natural spline (2 knots) | 3.00 | 1.05 | 1.02 | 1.08 | 56,004.17 |
| B-spline (50th, 90th percentile) | Natural spline (2 knots) | 3.00 | 1.06 | 1.00 | 1.13 | 56,025.59 |
| B-spline (10th, 75th, 90th percentile) | Natural spline (2 knots) | 3.00 | 1.07 | 1.00 | 1.13 | 56,028.76 |
| Linear | Integer | 7.00 | 1.06 | 1.02 | 1.10 | 55,305.60 |
| B-spline (50th, 90th percentile) | Integer | 7.00 | 1.08 | 1.00 | 1.17 | 55,346.45 |
| B-spline (10th, 75th, 90th percentile) | Integer | 7.00 | 1.07 | 1.00 | 1.15 | 55,351.50 |
| Linear | Strata lag0, lag1-3 | 7.00 | 1.06 | 1.02 | 1.10 | 55,298.03 |
| B-spline (50th, 90th percentile) | Strata lag0, lag1-3 | 7.00 | 1.08 | 1.00 | 1.17 | 55,311.85 |
| B-spline (10th, 75th, 90th percentile) | Strata lag0, lag1-3 | 7.00 | 1.08 | 1.00 | 1.17 | 55,316.41 |
| Linear | Natural spline (2 knots) | 7.00 | 1.06 | 1.02 | 1.10 | 55,299.50 |
| B-spline (50th, 90th percentile) | Natural spline (2 knots) | 7.00 | 1.09 | 1.00 | 1.17 | 55,311.85 |
| B-spline (10th, 75th, 90th percentile) | Natural spline (2 knots) | 7.00 | 1.09 | 1.00 | 1.17 | 55,320.31 |
