# Supplementary material for: Ambient temperature and mental health hospitalizations in Bern, Switzerland: A 45-year time-series study
Source: PLoS One. 2021 Oct 12;16(10):e0258302. doi: 10.1371/journal.pone.0258302 (PMC8509878; doi:10.1371/journal.pone.0258302)
Supplement: S1 Methods Appendix — (DOCX) [file pone.0258302.s009.docx]

# Description of the model selection process

Different models were tested by using a range of combinations of functions defined in the cross-basis term - the term used in distributed lag non-linear models (DLNMs) to model the exposure [23]. In the exposure-response dimension, a linear function and several non-linear functions (quadratic b-spline with knots at 50th and 90th percentile and at the 10th, 75th and 90th percentile of the observed temperature distribution) were used. In the lag-response dimension, different functions were considered: unconstraint, strata (lag 0, lag 1-3) and natural spline (two knots equally-spaced in the log scale). The number of lags was set to 3 and 7 days. These results, in terms of relative risks and its 95% confidence intervals, and quasi-Akaike Information Criterion (QAIC) for each model are shown in S3 Table. It can be observed that the model with linear function in the exposure-response dimension and unconstraint lag dimension with maximum lag of 3 provided the best fit. In S3 Figure, where a model with lag 7 is considered, it is shown that the association is mostly found in the lag0-3 - this supports our decision of reporting only for this window. In S4 Figure where the linear and the non-linear exposure-response (quadratic b-spline with knots at 50th and 90th percentile) of the temperature – hospitalizations associations are compared, it can be noticed that the curves are similar and the second curve (non-linear association) is almost linear.
