# Supplementary material for: Ambient temperature and mental health hospitalizations in Bern, Switzerland: A 45-year time-series study
Source: PLoS One. 2021 Oct 12;16(10):e0258302. doi: 10.1371/journal.pone.0258302 (PMC8509878; doi:10.1371/journal.pone.0258302)
Supplement: S2 Methods Appendix — (DOCX) [file pone.0258302.s010.docx]

The considered variables were several meteorological factors (relative humidity (%), precipitation (mm), atmospheric pressure (mmHg), atmospheric pressure difference (mmHg), sunshine duration (hours) and wind speed (km/h)) and air pollutants (NO2, PM10, and O3). These were included in the model separate models (with and without temperature) as linear function in the exposure-response dimension, unconstraint function modelling in the lag-response dimension. The number of lags considered was 3 for the weather variables and 1 for air pollutants, as suggested in previous literature. The association estimates for each of the confounders (with and without control for temperature), and temperature (controlled for each confounder) were extracted and compared (Fig S1 and S2). Note that air pollution data was only available for the period 1991-2018.

Among the tested variables, only wind speed and NO2 were associated with mental health hospitalizations, which seemed to be independent from temperature as their inclusion in the model did not affect the estimate of the latter (S1 and S2 Fig). Thus, any of them were considered as confounder in our analysis.
