# Supplementary material for: Ambient temperature and mental health hospitalizations in Bern, Switzerland: A 45-year time-series study
Source: PLoS One. 2021 Oct 12;16(10):e0258302. doi: 10.1371/journal.pone.0258302 (PMC8509878; doi:10.1371/journal.pone.0258302)
Supplement: Attachment — Submitted filename: Response to Reviewers.docx [file pone.0258302.s012.docx]

**General comments on the review**

We would like to thank once again the reviewers for their constructive comments and suggestions to improve the quality of our manuscript. We have carefully addressed all the points of the review in the current version of the manuscript and we have provided a detailed answer to each of the queries below.

**Comments from the editorial board**

The potential biological mechanisms regarding the effects of ambient temperature on mental health hospitalizations should be discussed in detail in the DISCUSSION section with enough references cited. Limitations of this study should also stated with details in the DISCUSSION section.

***We have included additional information in the discussion section on the biological mechanism explaining the effect of ambient temperature on mental health and, in particular, for schizophrenia and developmental disorders for which larger estimates were found.***

***Additionally, as suggested, we have extended the section on the study limitations in the discussion section.***

***The following references have been added to the manuscript:

17.	IPCC, 2021: Summary for Policymakers. In: Climate Change 2021: The Physical Science Basis. Contribution of Working Group I to the Sixth Assessment Report of the Intergovernmental Panel on Climate Change [MassonDelmotte, V., P. Zhai, et al. (eds.)]. Cambridge University Press. In Press.

21.	Butler R. The ICD-10 General Equivalence Mappings. Bridging the translation gap from ICD-9. Journal of AHIMA. 2007;78(9):84-5.***

***35. Ragettli MS, Röösli M. [Heat-health action plans to prevent heat-related deaths-experiences from Switzerland]. Bundesgesundheitsblatt, Gesundheitsforschung, Gesundheitsschutz. 2019;62(5):605-11.***

***42. Cianconi P, Betrò S, Janiri L. The Impact of Climate Change on Mental Health: A Systematic Descriptive Review. Front Psychiatry. 2020;11(74).***

***43. Maes M, Scharpé S, Verkerk R, D'Hondt P, Peeters D, Cosyns P, et al. Seasonal variation in plasma L-tryptophan availability in healthy volunteers. Relationships to violent suicide occurrence. Archives of general psychiatry. 1995;52(11):937-46.***

***48.	Dunn W. The impact of sensory processing abilities on the daily lives of young children and their families: A conceptual model. Infants and young children. 1997;9:23-35.***

***49.	Dunn W. The sensations of everyday life: empirical, theoretical, and pragmatic considerations. The American journal of occupational therapy : official publication of the American Occupational Therapy Association. 2001;55(6):608-20.***

***50.	Miller LJ, Anzalone ME, Lane SJ, Cermak SA, Osten ET. Concept evolution in sensory integration: a proposed nosology for diagnosis. The American journal of occupational therapy : official publication of the American Occupational Therapy Association. 2007;61(2):135-40.***

***51.	Tada K, Tateda H, Arashima S, Sakai K, Kitagawa T, Aoki K, et al. Follow-up study of a nation-wide neonatal metabolic screening program in Japan. A collaborative study group of neonatal screening for inborn errors of metabolism in Japan. European journal of pediatrics. 1984;142(3):204-7.***

***52.	Williams ZJ, Failla MD, Davis SL, Heflin BH, Okitondo CD, Moore DJ, et al. Thermal Perceptual Thresholds are typical in Autism Spectrum Disorder but Strongly Related to Intra-individual Response Variability. Scientific Reports. 2019;9(1):12595.***

***53.	Bolton M, Blumberg W, Ault L, Mogil H, Hanes S. Initial Evidence for Increased Weather Salience in Autism Spectrum Conditions. Weather, Climate, and Society. 2020;12.***

***54.	Scahill L, Lombroso PJ, Mack G, Van Wattum PJ, Zhang H, Vitale A, et al. Thermal sensitivity in Tourette syndrome: preliminary report. Perceptual and motor skills. 2001;92(2):419-32.***

***55.	Park RJ, Goodman J, Hurwitz M, Smith J. Heat and Learning. American Economic Journal: Economic Policy. 2020;12(2):306-39.***

***61.	Bouchama A, Dehbi M, Mohamed G, Matthies F, Shoukri M, Menne B. Prognostic factors in heat wave related deaths: a meta-analysis. Archives of internal medicine. 2007;167(20):2170-6.***

***62.	Davido A, Patzak A, Dart T, Sadier MP, Méraud P, Masmoudi R, et al. Risk factors for heat related death during the August 2003 heat wave in Paris, France, in patients evaluated at the emergency department of the Hôpital Européen Georges Pompidou. Emerg Med J. 2006;23(7):515-8.***

**Reviewer 1**

Review Recommendations:
1. This study used case-crossover design to compare different exposure periods for the same population. It needs a more detailed explanation for the hospitalization difference (figure2) caused by the conversion of icd9/icd10 and also for the possibility of underestimation and overestimation of its influence (association) resulted from the deviation.

***Thanks to the reviewer for the useful comment. We investigated further this issue and asked the hospital for further details about this increase in hospitalizations cases. Even though in this period there was a change in the classification of the disease, they considered that this was not the main reason for the increased number of mental health hospitalizations (contrary to what we stated in the previous review). In 1994, the management of the hospital took the administrative decision to shorten the length of stay for hospitalized patients. This could have led to an increase in incident hospitalizations cases. However, we would like to point that even though these changes in the length of stay could have altered the long-term trends of the outcome, this could have not introduced any bias in our estimations. As now clarified in the methods section, in the case-crossover design each day is compared to control days selected based on the day of the week, month and year. That is, for example, the exposure and outcome in the second Monday of January 1993 are compared with the exposure and outcome in all the other Mondays of January 1993. Thus, estimates reflect variations in the exposure-outcome happening on the short-term (within a month) and any temporal patterns occurring at longer time scales beyond that time frame are removed by design. This was clarified in the manuscript (lines 216-222: “Deviation from monotonicity in the long-term behaviour and change points can be also observed, as for (e.g. example the jump steep increase from in 1996 onwards). These, which could be possibly attributed to changes in diagnostic criteria or administrative decisions (e.g. reduction in the length of the stay which would allow for the admission of more patients). However, as mentioned in the method section, factors altering long-term temporal patterns would not introduce bias in the association estimates, as its potential confounding effect is removed by design”, lines 423-428).***

2. The explanation of patients' repeated hospitalization and disease severity is too brief.

***Thanks to the reviewer for raising this point. We have expanded the explanation for repeated hospitalizations and disease severity in the discussion section (lines 416-420).***

3. It should discuss more on the mechanism or the reason why developmental disorders and schizophrenia are affected by temperatures in the discussion section.

***We thank the reviewer for pointing out this important issue. We have included additional text in the discussion section on the mechanism of how ambient temperature affects schizophrenia and different developmental disorders (autism spectrum disorders, attention-deficit/hyperactivity disorder, tic syndromes and learning disabilities (lines 341-371).***

4. What are the possible reasons of that extremely high temperature (heatwave) are not significant? Please add in discussion section.

***We thank the reviewer for raising this issue. The non-significant effect of heatwaves can be explained by the low statistical power for this type of event in the study. Even though, heatwave occurrences have increased steadily in Switzerland in the last years, they still remain a rare event (with an average of 14-35 heatwave days per year (depending on the definition of heatwaves) (Table 2). We have added this explanation in the text (lines 303-305).***

5. The author stated that the risk is the same in warm and cold ranges, and with the same direction, magnitude and precision. (Response to Review1 Recommendations) How to come out this conclusion?

***Thanks for the comment, and we apologize if the statement was not clear in the previous review. As described in the manuscript, the association between temperature and the risk of hospitalizations was linear, thus the magnitude of the association (expressed as an increase in risk per 1C) is constant across the whole range of temperatures. Thus, according to our findings the risk of mental hospitalizations increased by 4% per 10C increase, regardless it is an increase from 15 to 25C, or -5 to 5C (i.e. warm and cold ranges).***
